# Astrocyte selective insulin receptor knockout in the 5xFAD model of amyloidosis

**DOI:** 10.1002/alz.095027

**Published:** 2025-01-09

**Authors:** Nicholas A Wright, Leopoldine B Galopin, Sophiya L Sims, Ruei‐Lung Lin, Edmund B Rucker, Colin B Rogers, Susan D Kraner, Christopher M. Norris, Olivier Thibault

**Affiliations:** ^1^ University of Kentucky College of Medicine, Lexington, KY USA; ^2^ University of Kentucky College of Medicine, Sanders‐Brown Center on Aging, Lexington, KY USA

## Abstract

**Background:**

We have recently published that overexpressing a constitutively active form of the insulin receptor beta subunit (IR‐β) in hippocampal neurons ameliorates spatial memory performance in the F344 rat model of aging (Frazier et al., 2020). Because astrocytes express IRs and are central to cellular energy and information transfer in the brain, here we focus on the knockdown of IR in astrocytes of the primary somatosensory cortex (S1) in the 5xFAD animal model. Using Cre‐lox recombination, we tested the cellular expression of AAV‐Cre recombinases and serotypes to selectively excise the IR, including 1. AAV5.GFAP.Cre.WPRE.hGH (AddGene), 2. AAV5.Gfa104.C1.Cre.WPRE.SV40 (UPenn), 3. AAV5.GFAP.GFP.Cre (UNC), 4. AAV8.GFAP.GFP.Cre (UNC; gift from Dr. Weikang Cai) and 5. AAV5.Gfa104‐Cre‐4×6T (Addgene/UPenn).

**Method:**

The 5xFAD and the IR^fl/fl^ were used to generate unique genotypes IR^fl/fl^; 5xFAD^−/−^ (WT) and IR^fl/fl^; 5xFAD^+/−^ (5XFAD). Mice were injected unilaterally in S1 with five different AAVs, followed with 3‐4 week recovery prior to the extraction and processing of the brain for immunohistochemical (IHC) and immunofluorescence (IF) analyses. Coronal sections of S1 were exposed to anti‐Cre recombinase primary antibodies labeled with secondary AlexaFluor antibodies. Counter stains used included DAPI and an anti‐GFAP or anti‐NeuN primary antibodies to address selectivity. In a second set of experiments conducted in older WT and 5xFAD mice injected unilaterally with AAV#2 and AAV#3 above, we used Western blotting techniques on two separate fractions (astrocyte‐depleted [AD] and astrocyte‐enriched [AE]). Samples were separated using the magnetic cell separation techniques followed by anti‐NeuN and anti‐GFAP antibodies to monitor selectivity.

**Result:**

Initial IHC indicates that the expression of the Cre recombinase under the GFAP promoter was surprisingly, mostly neuronal with very little expression in astrocytes. AAVs with the smaller Gfa104 promoter show improved specificity toward astrocytes (∼50%), with some expression in neurons. Our modified AAV shows higher cell‐type selectivity toward astrocytes.

**Conclusion:**

Overall, our attempt to target astrocyte IR knockdown has proven to be difficult and current efforts using molecular remodeling and targeting to astrocytes using an miRNA‐based AAV to degrade transcription in neurons, thus, enriching expression of Cre in astrocytes, is proving to be a very valuable strategy.

